# Experimental Infection of *Amblyomma aureolatum* Ticks with *Rickettsia rickettsii*

**DOI:** 10.3201/eid1705.101524

**Published:** 2011-05

**Authors:** Marcelo B. Labruna, Maria Ogrzewalska, João F. Soares, Thiago F. Martins, Herbert S. Soares, Jonas Moraes-Filho, Fernanda A. Nieri-Bastos, Aliny P. Almeida, Adriano Pinter

**Affiliations:** Author affiliations: University of São Paulo, São Paulo, Brazil (M.B. Labruna, M. Ogrzewalska, J.F. Soares, T.F. Martins, H.S. Soares, J. Moraes-Filho, F.A. Nieri-Bastos, A.P. Almeida);; Superintendency of Control of Endemic Diseases, São Paulo (A. Pinter)

**Keywords:** bacteria, vector-borne infections, Rickettsiae, **s**potted fever, Rickettsia rickettsii, Amblyomma aureolatum, ticks, transovarial transmission, transstadial maintenance, research

## Abstract

We experimentally infected *Amblyomma aureolatum* ticks with the bacterium *Rickettsia rickettsii*, the etiologic agent of Rocky Mountain spotted fever (RMSF). These ticks are a vector for RMSF in Brazil. *R. rickettsii* was efficiently conserved by both transstadial maintenance and vertical (transovarial) transmission to 100% of the ticks through 4 laboratory generations. However, lower reproductive performance and survival of infected females was attributed to *R. rickettsii* infection. Therefore, because of the high susceptibility of *A. aureolatum* ticks to *R. rickettsii* infection, the deleterious effect that the bacterium causes in these ticks may contribute to the low infection rates (<1%) usually reported among field populations of *A. aureolatum* ticks in RMSF-endemic areas of Brazil. Because the number of infected ticks would gradually decrease after each generation, it seems unlikely that *A. aureolatum* ticks could sustain *R. rickettsii* infection over multiple successive generations solely by vertical transmission.

The bacterium *Rickettsia rickettsii* is the etiologic agent of the deadliest rickettsiosis, Rocky Mountain spotted fever (RMSF), which is referred to as Brazilian spotted fever in Brazil (*1*). The distribution of *R. rickettsii* is restricted to the Americas; confirmed cases of RMSF have been reported in Canada, United States, Mexico, Costa Rica, Panama, Colombia, Brazil, and Argentina. Different tick species have been implicated as vectors of *R. rickettsii* in different geographic areas. Whereas the ticks *Dermacentor andersoni* and *D. variablilis* are the main vectors in the United States, the *Amblyomma cajennense* tick is the most common vector in South America (*1**,**2*). The tick *Rhipicephalus sanguineus* has also been implicated as a vector for *R. rickettsii* in a few areas in Mexico and the state of Arizona in the United States (*2**,**3*). However, *A. aureolatum* ticks are the main vector in the metropolitan area of São Paulo, Brazil, distinguishing this area from the remaining RMSF-endemic areas of Latin America (*1**,**4**,**5*).

Adult *A. aureolatum* ticks feed chiefly on Carnivora species (mostly domestic dogs), but immature ticks (larvae, nymphs) prefer to feed on passerine birds and a few rodent species (*6*). Humans have reported being attacked only by adults, usually by a single tick (*7*), because population density of *A. aureolatum* ticks is usually low (*8*). One field study in an RMSF-endemic area of São Paulo found that <1% of the *A. aureolatum* adult ticks were infected by *R. rickettsii* (*5*). The reasons for such a low infection rate are not clear; a recent laboratory study reported that up to 100% of *A. aureolatum* larvae efficiently acquired and maintained the *R. rickettsii* infection to the nymphal stage, after a larval feeding on experimentally infected guinea pigs (*9*). Therefore, we evaluated the transstadial maintenance and transovarial transmission of *R. rickettsii* in *A. aureolatum* ticks through 4 consecutive generations of this tick in the laboratory; the vector competence of larvae, nymphs, and adults; and deleterious effects of *R. rickettsii* on the survival of larvae and nymphs and on the reproductive performance of female ticks.

## Materials and Methods

In a laboratory experiment previously reported (*9*), the first generation (F_1_) larval progeny of engorged *A. aureolatum* female ticks collected in Atibaia, São Paulo State, Brazil, were allowed to feed on 4 *R. rickettsii–*infected guinea pigs (infected group) and 2 uninfected guinea pigs (control group). These guinea pigs were infected by intraperitoneal inoculation of a homogenate of *R. rickettsii*–infected guinea pig organs, as detailed previously (*9*). The Taiaçu strain of *R. rickettsii* used in this experiment was the first guinea pig passage from a naturally infected *A. aureolatum* tick, which was cryopreserved before this strain was adapted for in vitro growth (*5*). Engorged tick larvae obtained from the infected and control groups of guinea pigs were held in an incubator at 23°C and 90% relative humidity for molting to nymphs. As reported (*9*), 100% of the resultant nymphs from the infected group were shown to be infected by *R. rickettsii,* whereas no nymph from the control group was infected by rickettsiae. These F_1_ nymphs were simultaneously used for the study we report here.

The F_1_ unfed nymphs of infected and control groups were reared separately in the laboratory for 4 consecutive generations until they were F_4_ unfed adults. Throughout the experiment, infestations with infected ticks and control groups were done in parallel; infested animals were held in the same room under the same environmental conditions as the controls. Male guinea pigs and female rabbits were infested with larvae and nymphs, and dogs and rabbits were infested with adult ticks. However, in each infestation with a given tick stage, different individuals of the same host species were used at the same time for infected and control groups. Every guinea pig or rabbit used in this study was tick naive; these animals were provided by a private laboratory that raised the animals under proper sanitary conditions. The dogs used for adult infestations had been infested by *A. aureolatum* ticks in a previous study (*10*), when they were also infected with *R. rickettsii*; the dogs were therefore immune to RMSF. Infestation of each animal was performed inside a feeding chamber glued to its shaved dorsum, as previously described (*11*). All infested animals had their temperature rectally measured daily from the day of infestation (day 0) to 21 days postinfestation. When present, skin lesions (e.g., scrotal reactions) during this period were also noted. Naturally detached engorged larvae, nymphs, or female ticks were recovered daily from the feeding chambers of the infested animals of both groups and immediately taken to a single incubator adjusted to 23°C and 95% relative humidity for molting (for engorged larvae and nymphs) or for egg laying and incubation (for engorged females). Engorged females had their individual weight measured the day they detached from the host. In addition, the total egg mass deposited by each female was weighed on the day of the end of oviposition and a conversion efficiency index (CEI = mg egg mass/mg engorged female × 100), which measures the efficiency with which a female tick converts body weight into eggs (*12*), was determined for each female that oviposited. Percentage of egg hatching for each egg mass was visually estimated (*13*).

During the experiment, random samples of unfed F_1_ nymphs and adults; F_2_ eggs and nymphs; and F_4_ eggs, larvae, nymphs, and adults from both infected and control groups were submitted for DNA extraction as described (*9*) and tested by PCR targeting a 401-bp fragment of the rickettsial *gltA* gene, as described (*9*). Eggs were tested in pools (1 egg pool containing 5–10 eggs/female), whereas larvae, nymphs, and adults were tested individually. At the end of the study, PCR products from 3 F_4_ nymphs underwent DNA sequencing (*5*), and the resultant sequence was compared with GenBank data by BLAST analysis (www.ncbi.nlm.nih.gov/blast).

All tick-infested guinea pigs and rabbits were tested for seroconversion to *R. rickettsii* antigens. For this purpose, blood samples were collected at 0 days postinfestation and 21 days postinfestation; these samples were tested for anti–*R. rickettsii* reactive antibodies by immunofluorescence assay (IFA), as previously described (*14*). Some infested guinea pigs that died before the 21 days postinfestation were not tested by IFA because a second blood sample was not obtained; however, a fragment of their lungs was submitted to DNA extraction by using the DNeasy Tissue Kit (QIAGEN, Chatsworth, CA, USA) and tested by the same PCR protocol referenced above.

During the experiment, tick biologic parameters were compared between infected and control groups. For this purpose, larval and nymphal molting success and female oviposition success (i.e., death of engorged ticks) were compared by the χ^2^ test. In addition, weight of engorged females and their corresponding egg masses, percentage of egg hatching, and CEI values were compared by Student *t* test. Values were considered significantly different when p<0.05. The study was approved by the Bioethical Committee in Animal Research of the Faculty of Veterinary Medicine of the University of São Paulo (protocol no. 1644/2009).

## Results

The infected tick group remained infected by rickettsiae through 4 consecutive generations, until the end of the experiment (F_4_ unfed adults). In all infestations performed with ticks from this group, fever developed in all guinea pigs and rabbits 5–9 days postinfestation. Guinea pigs also had scrotal reactions characterized by swelling, followed by necrosis in most animals. Among the 16 guinea pigs infested with infected larvae or nymphs, 8 died during the febrile period. Of the 8 dead guinea pigs, 6 had their lungs tested by PCR, which detected rickettsial DNA. The remaining guinea pigs plus the rabbits showed seroconversion for *R. rickettsii* at 21 days postinfestation (Table 1). In contrast, no guinea pig or rabbit infested with ticks from the control group experienced fever or seroconverted; i.e., they were nonreactive for *R. rickettsii* at both 0 days postinfestation and 21 days postinfestation. Fever did not develop in the dogs infested with generations F_1_ and F_3_ adult ticks, and serum was not tested on 0 days postinfestation and 21 days postinfestation because it was already known that the dogs were seroreactive to *R. rickettsii* because they had been previously infected in another study (*10*).

**Table 1:** Clinical data of hosts infested by a *Rickettsia rickettsii*–infected colony of *Amblyomma aureolatum* ticks, and results of PCR performed on unfed ticks from 4 consecutive generations (F_1_ to F_4_)*

Tick stage and generation	Infested hosts							PCR of unfed ticks, no. infected/no. tested
	Host, no.	No. ticks/host	Fever†		No. deaths	Diagnostic test		
			No.	Onset, dpi		PCR‡	IFA§	
Nymph–F_1_	Guinea pig, 2	50	2	6	2	Pos	ND	45/45¶
Adult–F_1_	Dog, 2	20 couples	0	–	0	ND	ND	10/10
Larva–F_2_	Guinea pig, 2	1,000–2,000	2	7–8	1	ND	Pos	–
Larva–F_2_	Rabbit, 3	1,000–2,000	3	7–9	0	ND	Pos	–
Nymph–F_2_	Guinea pig, 3	80	3	5	2	Pos	Pos	30/30
Nymph–F_2_	Rabbit, 1	200	1	6	0	ND	Pos	–
Adult–F_2_	Rabbit, 2	7 couples	2	7–8	0	ND	Pos	–
Larva–-F_3_	Guinea pig, 4	1,000–2,000	4	7–8	2	Pos	Pos	–
Nymph–F_3_	Rabbit, 1	400	1	6	0	ND	Pos	–
Adult–F_3_	Dog, 1	12 couples	0	–	0	ND	ND	–
Larva–F_4_	Guinea pig, 5	1,000–3,000	5	6–7	1	ND	Pos	100/100
Nymph–F_4_	Rabbit, 1	500	1	5	0	ND	Pos	30/30

*dpi, days postinfestation; IFA, immunofluorescence assay; pos, positive; ND, not done; –, data not collected. †Rectal temperature >40°C. ‡PCR performed on lung-extracted DNA from guinea pigs that died during the febrile period. §A positive result means seroconversion, namely nonreactive (titer <64) at 0 DPI, and reactive (titer >1,024) at 21 dpi. Previously reported by Labruna et al. (*9*).

All PCRs performed on unfed ticks from the infected group resulted in amplicons compatible with *R. rickettsii.* These amplicons resulted from 45 F_1_ nymphs, 10 F_1_ adults (5 males, 5 females), 30 F_2_ nymphs, 100 F_4_ larvae, 30 F_4_ nymphs, and 6 F_4_ adults (3 males, 3 females), which were all tested individually (Table 1). In addition, 12 F_2_ egg pools and 5 F_4_ egg pools, derived from all F_1_ and F_3_ infected engorged females, also yielded PCR amplicons compatible with *R. rickettsii.* The 100 PCR-positive F_4_ larvae cited above encompassed 5 groups of 20 larvae, each derived from a different F_3_ engorged female. PCR products from 3 F_4_ nymphs underwent DNA sequencing, and the resultant sequence was 100% identical to *R. rickettsii* strain Taiaçu, available in GenBank (accession no. DQ115890).

For the control group, no visible amplicon was generated by all ticks tested by PCR, which included 20 F_1_ nymphs, 6 F_1_ adults (3 males, 3 females), 10 F_2_ nymphs, 30 F_4_ larvae, 20 F_4_ nymphs, and 6 F_4_ adults (3 males, 3 females), which were all tested individually, and 13 F_2_ egg pools and 6 F_4_ egg pools, derived from all F_1_ and F_3_ control engorged females. The 30 PCR-negative F_4_ larvae cited above encompassed 3 groups of 10 larvae, with each group derived from a different F_3_ engorged female.

Although the mortality rate for engorged F_4_ larvae and F_3_ nymphs was higher for the infected group than the control group, overall molting success of engorged larvae and nymphs was similar between infected and control ticks because there was no significant difference in the molting success of these ticks when the 4 generations were grouped. However, there was an overall lower egg-laying success of engorged females from the infected group when compared with the control group (Table 2); i.e., although 89.7% of the control engorged females successfully oviposited in the incubator, only 66.7% of the infected females oviposited in the same incubator. Regarding the reproductive performance of these females, a few significant differences were observed between infected and control females, generally in favor of control ticks (Table A1).

**Table 2:** Molting and oviposition success of *Amblyomma aureolatum* ticks infected by *Rickettsia rickettsii* (infected group) and noninfected (control group) through 4 consecutive generations (F_1_ to F_4_) in the laboratory*

Tick generation	No. larvae that molted to nymphs/ no. recovered engorged larvae (% molting success)			No. nymphs that molted to adults/ no. recovered engorged nymphs (% molting success)			No. females that oviposited/ no. recovered engorged females (% oviposition success)	
Infected	Control	Infected	Control	Infected	Control
F_1_	632/679 (93.1)	226/250 (90.4)		68/71 (95.8)	25/29 (86.2)		12/16 (75)	13/13 (100)
F_2_	300/349 (86.0)	791/959 (82.5)		57/60 (95.0)	24/28 (85.7)		5/10 (50)	7/9 (77.8)
F_3_	292/443 (65.9)	737/1,179 (62.5)		49/110 (44.5)	51/68 (75.0)†		5/7 (71.4)	6/7 (85.7)
F_4_	721/1,278 (56.4)	868/1,358 (63.9)†		138/148 (93.2)	46/52 (88.5)		–	–
Total	1,945/2,749 (70.7)	2,622/3,746 (70.0)		312/389 (80.2)	146/177 (82.5)		22/33 (66.7)	26/29 (89.7)†

*–, data not collected. †Molting or oviposition success values for infected and control ticks of the same tick stage were significantly different (p<0.05).

## Discussion

In the study reported here, *R. rickettsii* was preserved by transstadial maintenance and transovarial transmission in *A. aureolatum* ticks for 4 consecutive generations, because all tested eggs, larvae, nymphs, and adults from the infected group were shown by PCR to contain rickettsial DNA, from the first to the fourth generation. In addition, infestations of guinea pigs and rabbits with larvae and nymphs from the 4 generations and adults from the third generation confirmed that ticks of these 3 developmental stages from the 4 generations were infected by *R. rickettsii* because all infested guinea pigs and rabbits became infected by *R. rickettsii,* which was confirmed by seroconversion through IFA or PCR in addition to compatible clinical data. These results also confirm that larvae, nymphs, and adults of *A. aureolatum* ticks are competent vectors of *R. rickettsii.* Together, our results strongly support clinical and epidemiologic data that have implicated the *A. aureolatum* tick as the main vector of *R. rickettsii* in the metropolitan area of São Paulo (*4**,**5**,**9**,**15*).

All infected egg pools tested by PCR yielded rickettsial DNA, indicating a transovarial transmission rate (the proportion of infected females giving rise to at least 1 infected egg or larva) of 100% among *R. rickettsii*–infected females. Because all the individual larvae from the infected group tested by PCR also yielded rickettsial DNA, a filial infection rate (proportion of infected eggs or larvae obtained from an infected female) of 100% is also likely to have occurred. In contrast, no egg pool or individual larva from the control group yielded rickettsial DNA by PCR. These results are in agreement with the larval infestations, which resulted in confirmed rickettsiosis in all guinea pigs or rabbits infested by larvae from the infected group and with no rickettsiosis in animals infested with control group larvae. Although transovarial transmission of spotted fever rickettsiae in ticks seems to occur worldwide (*16*), including *R. rickettsii* in New World ticks (*17**–**23*), few studies have quantified this perpetuation route. Previous studies in the United States demonstrated a 100% transovarial transmission rate and a 100% filial infection rate of *R. rickettsii* in *D. variabilis* naturally infected female ticks and in *D. andersoni* female ticks, either naturally infected or experimentally infected during the larval stage (*20**,**24*).

In the study reported here, although F_2_ female ticks fed on susceptible rabbits, F_1_ and F_3_ females fed on dogs previously infected by *R. rickettsii.* As expected, these dogs showed no clinical signs during the study, a condition certainly related to their immune status because they were shown to have high antibody titers against *R. rickettsii* (data not shown). Regardless of feeding on immune (dogs) or susceptible (rabbits) hosts, 100% transovarial transmission rates and filial infection rates were found for all generations evaluated. Similarly, Burgdorfer and Brinton (*24*) observed 100% transovarial transmission rates and filial infection rates for *D. andersoni* female ticks that fed on either immune or susceptible hosts, resulting in no alteration of biological characteristics of the bacterium. Thus, the host immune status to *R. rickettsii* does not seem to alter the transovarial transmission of *R. rickettsii* in ticks.

Overall, no expressive differences in mortality rates were observed between engorged immature ticks from the infected and control groups. These results contrast with those of a previous study (*21*) that reported much higher mortality rates for infected *D. andersoni* engorged larvae and nymphs (34.9%–97.7%) than those observed for uninfected sibling ticks (1.4%–2.1%) and also with those of experimental studies on *R. conorii* in *R. sanguineus* ticks, in which significant mortality rates of immature ticks were observed when compared with uninfected ticks (*25**,**26*). On the other hand, our results demonstrated that the mortality of *R. rickettsii*–infected engorged females was 3× higher than the mortality of uninfected females; i.e., oviposition success was only 66.7% in the former and 89.7% in the latter. In addition, the reproductive performance of uninfected females was significantly higher than that of infected females, as demonstrated by higher egg mass weight for control ticks. Because both the infected and control ticks were siblings derived from the same field-engorged females used to start a laboratory colony, reared under the same laboratory conditions during the whole study, we conclude that the lower survival and reproductive performance of infected females was a result of a deleterious effect caused by the *R. rickettsii* infection. These results are in agreement with those of a previous study (*24*), which reported higher mortality of *R. rickettsii*–infected *D. andersoni* and *D. variabilis* engorged female ticks and lower egg masses oviposited by these females than by uninfected females. This higher mortality and lower egg mass was attributed to massive rickettsial development in the female body, including the ovaries (*24*). Tick mortality is much more influential on the tick population when it occurs in engorged females; i.e., although each dead egg, larva, or nymph is only less 1 subsequent larva, nymph, or adult, respectively, in the tick population, a dead engorged female represents thousands of eggs fewer in the following generation.

Different field studies in Brazil and in the United States have reported that <1% of the *Dermacentor* spp. and *A. aureolatum* ticks, respectively, are found naturally infected by *R. rickettsii* within RMSF-endemic areas (*1**,**5**,**27*). On the other hand, the present study demonstrated that besides being highly susceptible to *R. rickettsii* infection, *A. aureolatum* ticks are highly efficient in maintaining the infection through 100% transstadial transmission, transovarial transmission, and filial infection rates. The main reason for these contrasting findings is the deleterious effect the *R. rickettsii* infection causes in ticks, as previously demonstrated for *Dermacentor* spp. ticks (*21**,**24**,**27*). Therefore, despite of the high susceptibility of *A. aureolatum* ticks to *R. rickettsii* infection, the higher mortality and reduced reproductive performance of infected engorged females may contribute to low infection rates among *A. aureolatum* tick field populations in RMSF-endemic areas of the São Paulo metropolitan area, such as the 0.9% infection rate previously reported (*5*).

On the basis of our results, it seems unlikely that *A. aureolatum* could sustain *R. rickettsii* infection over multiple successive generations solely by vertical transmission because the number of infected ticks would gradually decrease after each generation. Thus, horizontal transmission through the participation of amplifier vertebrate hosts in the formation of new lineages of infected ticks seems to be crucial for maintenance of *R. rickettsii* in the RMSF-endemic areas where the *A. aureolatum* tick is implicated as the principal vector, just as has been reported for RMSF-endemic areas in the United States, where *Dermacentor* spp. ticks are the vector (*21**,**27*). Although natural amplifier hosts are known for *Dermacentor* spp. ticks in RMSF-endemic areas in the United States (*27*), and for *A. cajennense* ticks in areas endemic for Brazilian spotted fever in Brazil (*1*), they are not known for *A. aureolatum* ticks. Further studies should test the natural hosts of *A. aureolatum* immature tick stages, which are some passerine birds and small rodents (*6*), for their competence to act as amplifier hosts of *R. rickettsii* to *A. aureolatum* ticks. Regarding dogs, the main host for the adult stage of *A. aureolatum* ticks within the São Paulo metropolitan area, even though it has been shown that dogs are capable of having rickettsial infections sufficient to infect other tick species (i.e., they are a competent amplifier host) (*23*), only adult-stage *A. aureolatum* ticks feed on dogs. Because transovarial transmission rates are likely to be low or absent when the primary infection of ticks occurs during adult feeding (*18**,**23**,**24*), the epidemiologic influence of dogs as amplifier hosts of *R. rickettsii* for *A. aureolatum* ticks is questionable. Therefore, future studies should target potential hosts of the immature stages of *A. aureolatum* ticks that could act as amplifier hosts.

## Appendix

**Table A1:** Reproductive data of *Amblyomma aureolatum* engorged female ticks infected by *Rickettsia rickettsii* (infected group) and noninfected (control group) through 3 consecutive generations in the laboratory*

Tick generation	Engorged weight, mg			Egg mass weight, mg			% Egg hatching			CEI	
	Infected	Control		Infected	Control		Infected	Control		Infected	Control
F_1_	849.0 ± 218.5 (375.2–1,220.9)	968.2 ± 258.8 (509.0–1,517.3)		278.0 ± 129.3 (60.2–466.2)	430.1 ± 185.6† (152.1–703.0)		29.3 ± 27.7 (0–90)	13.5 ± 9.1 (1–40)		34.7 ± 13.4 (5.9–51.2)	43.9 ± 12.9 (22.0–61.0)
F_2_	740.0 ± 203.9 (332.5–1,034.2)	474.6 ± 197.9† (198.0–856.4)		141.0 ± 113.0 (23.1–338.9)	221.3 ± 94.9 (60.7–314.0)		71.0 ± 37.2 (0–100)	74.3 ± 27.7 (30–100)		21.0 ± 16.5 (5.2–50.2)	43.5 ± 7.2† (30.6–51.8)
F_3_	615.8 ± 138.2 (410.0–790.0)	1045.3 ± 118.5† (890.0–1,213.0)		337.5 ± 138.3 (189.0–487.0)	413.6 ± 241.6 (122.0–740.0)		62.0 ± 27.1 (20–100)	47.0 ± 39.6 (1–95)		52.3 ± 6.5 (44.1–61.6)	37.8 ± 20.5 (13.3–61.0)
Total	766.5 ± 219.4 (332.5–1,220.9)	833.6 ± 323.3 (198.0–1,517.3)		260.4 ± 138.6 (23.1–487.0)	370.1 ± 203.2† (60.7–740.0)		46.2 ± 35.4 (0–100)	37.6 ± 35.8 (1–100)		35.6 ± 16.8 (5.2–61.6)	42.4 ± 14.1 (13.3–61.0)

*Values are mean ± standard error (range). CEI, conversion efficiency index = egg mass weight/female engorged weight × 100. †Significantly different (p<0.05) values between infected and control groups for each tick biologic parameter.
